# Alterations in the Expression of a Set of miRNAs in Endometrial Cancer and Their Correlation with Clinical Variables and the p53 Signaling Pathway

**DOI:** 10.3390/ijms26115215

**Published:** 2025-05-29

**Authors:** Jessica Alejandra Zapata García

**Affiliations:** Health Sciences Decan, Universidad Autonoma de Guadalajara, Zapopan 45129, Mexico; genetica860@gmail.com; Tel.: +52-332-287-26-73

**Keywords:** endometrial cancer, hsa-miR-182, hsa-miR-449a, hsa-miR-760, overall survival, relative risk, diagnosis, p53

## Abstract

Endometrial cancer is the fifth most common cancer worldwide, with one of the highest incidence and mortality rates. Its incidence is projected to increase 55% by 2030. Currently, the techniques used for its detection are heterogeneous and can be invasive and nonspecific. In this context, omics studies have gained relevance, providing solutions that have improved patient diagnosis and prognosis. In this study, we used data from the GSE268888 study as discovery cohort and data from the TCGA consortium as a validation cohort. Expression analyses were performed to identify miRNAs overexpressed in endometrial cancer. These miRNAs were then analyzed in relation to diagnostic and prognostic clinical variables. The target genes of these miRNAs were identified using bioinformatic tools, and functional enrichment analyses were conducted with this gene set to explore their involvement in relevant oncogenic signaling pathways. We also calculated the structural topology of the miRNA–target complexes and computed their correlation coefficients. We found that hsa-miR-182 and hsa-miR-760 had diagnostic and prognostic relevance and interacted with the p53 signaling pathway. Specifically, hsa-miR-449a was associated with diagnosis, but not with prognosis. Furthermore, we found that these miRNAs share *TP53INP1* as a common target gene and estimated a high probability of complex formation, along with a positive correlation between these miRNAs and *TP53INP1* in more advanced stages of the disease. These findings suggest that this miRNA signature has potential to be used as a diagnostic and prognostic biomarker and could serve as a foundation for future therapeutic strategies for endometrial cancer. However, further experimental validation is needed to confirm its clinical applicability.

## 1. Introduction

According to global statistics, endometrial cancer (EC) ranks as the fifth most common malignancy among the top fifteen cancer types, exhibiting some of the highest incidence and mortality rates worldwide, with rates of 14.1 and 7.1, per 100,000 individuals, respectively [1]. It is incidence is projected to increase 55% by 2030 [2].

Currently, there is no consensus regarding its detection. Common diagnostic techniques include transvaginal ultrasound, hysteroscopy, or endometrial biopsy, which can be expensive, invasive, or have low specificity [3]. Furthermore, when EC is diagnosed at advanced stages (II, III, and IV), patient prognosis tends to be poor [4].

In response to these challenges, microRNAs (miRNAs or miRs) have gained relevance. These small molecules are derived from DNA, and can interact with coding and non-coding regions, or with the promoter regions of their target messenger RNAs (mRNAs), leading to effects such as repressing or activating transcription and translation [5]. MicroRNAs can mediate intercellular communication by being transported through microvesicles or exosomes [6]. In cancer, they have been shown to regulate key biological processes such as cell proliferation, apoptosis, and invasion [7].

In EC, miRNAs have been reported to regulate the expression of genes involved in disease pathogenesis [8]. The dysregulation of specific miRNAs, such as miR-10b, miR-21, and miR-135a (upregulated) and miR-29c, miR-34a, and miR-145 (downregulated), has been associated with primary tumor growth. Additionally, elevated levels of miR-19a and reduced expression of miR-126, miR-133a, and miR-503 contribute to the formation of the pre-metastatic niche [9]. miRNAs have also been shown to modulate key oncogenic signaling pathways implicated in EC, including JAK/STAT, EGFR, TGF-β, and p53 [10].

In clinical practice, miRNAs have become a useful tool for the diagnosis and prognosis of EC. Since they can be easily detected [4], and offer a cost-effective and minimally invasive option, small RNA sequencing analysis currently represents one of the best strategies for quantifying the presence of miRNAs in samples from EC patients with high sensitivity and specificity [11].

The aim of this study was to identify differentially expressed miRNAs (DEMs) in a cohort of patients with EC and to validate those with high expression using data from The Cancer Genome Atlas (TCGA) consortium as a validation cohort. We further evaluated the diagnostic and prognostic capacity of these miRNAs by assessing their sensitivity, specificity, and associations with overall survival and relative risk. Additionally, we also applied enrichment analysis to identify potential molecular mechanisms involving the target genes of each of the DEMs and modeled the likelihood of 2D and 3D structural formations of the corresponding miRNA–target complexes.

## 2. Results

### 2.1. Differentially Expressed miRNAs in Endometrial Cancer

**Figure 1:**
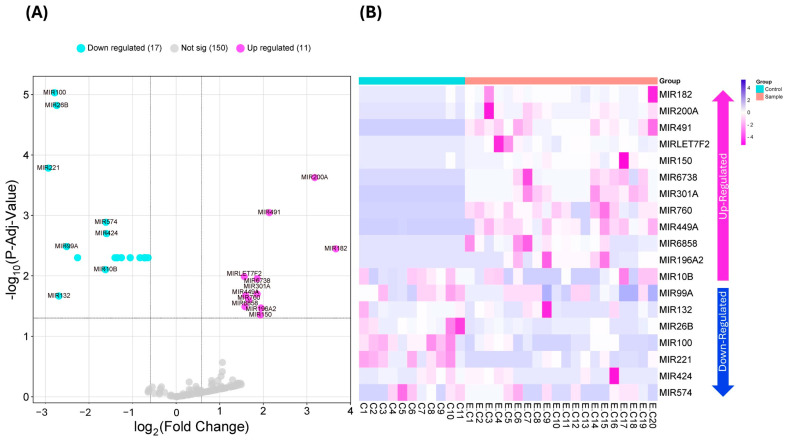
Differentially expressed miRNAs in endometrial cancer. (**A**) Volcano plot showing the distribution of differentially expressed miRNAs. The normalized adjusted *p*-value is represented as the negative base-10 logarithm (−log10 P-Adj-Value), while the normalized fold change is expressed as the base-2 logarithm (log2 Fold Change). miRNAs were filtered based on a P-Adj-Value < 0.05. Overexpressed miRNAs are shown in fuchsia, while underexpressed genes are shown in light blue. (**B**) Heatmap illustrating the fold change in the 19 differentially expressed miRNAs (DEMs) identified in endometrial cancer samples. DEMs were selected based on a fold change ≥ 1.5 and a P-Adj-Value ≤ 0.05. C1–11: Non-cancerous endometrial samples (light blue bar, top left). EC1–20: Endometrial cancer samples (pink bar, top right).

This study identified DEMs by comparing 20 endometrial cancer patients with 11 healthy endometrial samples from the study GSE268888. Clinical data for the samples are available in the supplementary material of the work by Hegde et al. [12]. According to our cutoff criteria (Fold Change ≥ 1.5 and adjusted *p*-value ≤ 0.05), a total of 19 miRNAs were identified, including 11 upregulated miRNAs and 8 downregulated miRNAs, as shown in the volcano plot presented in Figure 1A. The FPKM values were obtained from DESeq2 for each miRNA in the control and endometrial cancer samples and plotted in the heatmap presented in Figure 1B.

### 2.2. Validation of Differentially Expressed miRNAs in Endometrial Cancer Subtypes

After identifying the DEMs, we aimed to validate the expression of upregulated miRNAs in a larger cohort of samples and determine whether differences were present across the different stages of endometrial cancer. To achieve this, we analyzed data from 501 endometrial cancer samples and 31 control samples from the TCGA consortium study.

**Figure 2:**
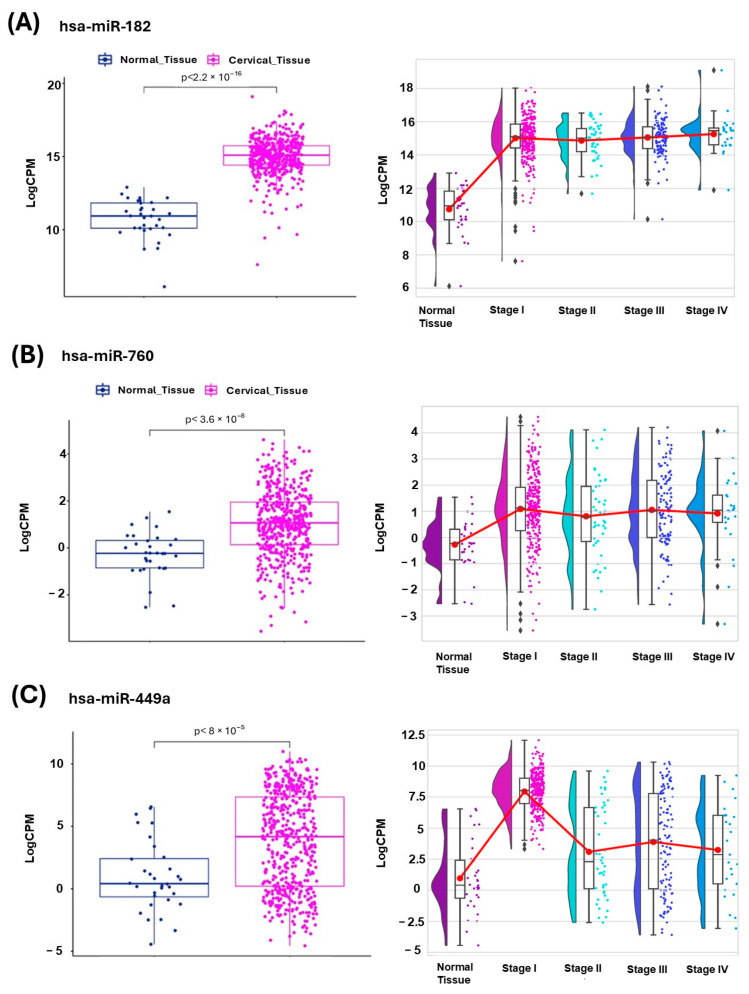
Boxplots of the expression of three miRNAs in the TCGA dataset. The boxplots on the left side of each panel (**A**–**C**) represent the expression levels of miRNAs in samples obtained from cancerous tissue (shown in fuchsia) and non-pathological tissue (shown in blue). Each point represents the miRNA expression level of an individual patient. The *p*-value reflects the result of the nonparametric Wilcoxon test comparing normal and cancerous tissue samples. The right side of each panel (**A**–**C**) shows the expression of miRNAs in endometrial cancer stages I, II, III, and IV compared to normal endometrial tissue. The red line represents the mean expression trend across samples. Counts per million (CPM) and log-transformed gene expression change with base 2 (Log2FC) are displayed. (**A**) hsa-miR-182, (**B**) hsa-miR-760, and (**C**) hsa-miR-449a.

We found that three of the eleven upregulated DEMs exhibited higher expression in endometrial cancer samples compared to normal endometrial tissue, as shown in the boxplots on the left side of panels A, B, and C of Figure 2. Regarding expression changes across stages, we found that hsa-miR-182 and hsa-miR-760 maintained a trend of higher expression in stages I, II, III, and IV (right side of panels A and B, respectively), this difference being most evident in the case of hsa-miR-182. In particular, hsa-miR-449a exhibited higher levels in stage I, than in stages II, III, and IV.

### 2.3. miRNAs with Diagnostic and Prognostic Value in Endometrial Cancer

**Figure 3:**
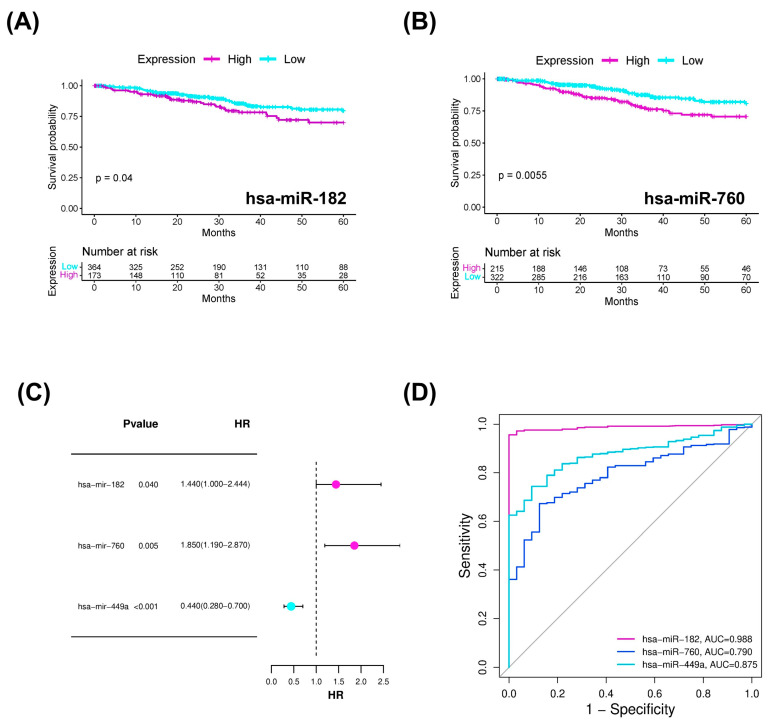
Overall survival, forest plot, and ROC curves of overexpressed miRNAs in endometrial cancer. Panels (**A**,**B**) present the overall survival curves and *p*-values obtained using the log-rank test and Cox regression model for hsa-miR-182 and hsa-miR-760, respectively. At the bottom of each panel, the number of patients at risk is shown over time (in months). Panel (**C**) displays a forest plot of the univariate Cox regression analysis, illustrating the hazard ratios (HR) with their respective 95% confidence intervals (95% CI) and *p*-values for hsa-miR-182, hsa-miR-760, and hsa-miR-449a. Each point represents the estimated effect size for an individual study. Panel (**D**) represents the area under the curve (AUC) of the ROC curves based on the expression levels of these three miRNAs.

To determine whether the overexpressed DEMs are clinically relevant, we performed survival analyses. These analyses showed that the elevated expression of hsa-miR-182 and hsa-miR-760 correlates with a lower probability of disease-free survival, unlike hsa-miR-449a (Figure 3A,B). Furthermore, Figure 3C presents the hazard ratio (HR) calculations, which confirm the trend for these two miRNAs to predict a worse prognosis for patients with endometrial cancer when their expression is elevated, but this is not the case for hsa-miR-449a (HR < 1). Regarding the receiver operating characteristic (ROC) curve analysis, the area under the curve (AUC) values for the four miRNAs were above the midline, with hsa-miR-182 presenting the highest value (AUC: 0.988), followed by hsa-miR-449a (AUC: 0.875), and hsa-miR-760 (AUC: 0.790). Therefore, all miRNAs effectively discriminated between endometrial cancerous and non-cancerous endometrial samples, with hsa-miR-182 showing the highest sensitivity (95%) and specificity (99%).

### 2.4. Target Prediction and Molecular Analysis

**Figure 4:**
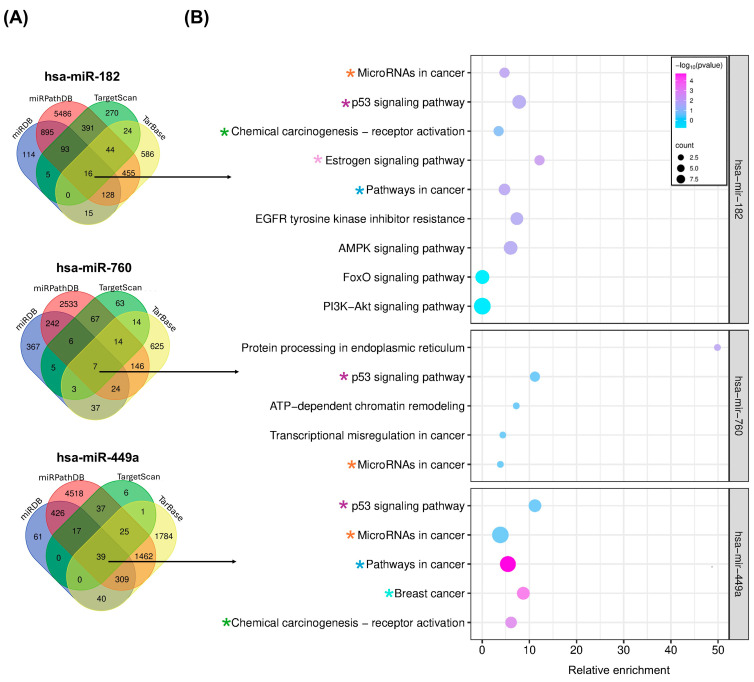
Targets of miRNAs and enrichment analysis. (**A**) Venn diagrams illustrating the overlap of target genes for hsa-miR-182, hsa-miR-760, and hsa-miR-449a. The arrow indicates the number of common target genes identified in the miRDB, miRPathDB, TargetScan, and TarBase databases. (**B**) Signaling pathways significantly enriched with miRNA target genes. The color scale represents the relative enrichment, while the size of the circles indicates the number of enriched genes in each pathway. The x-axis of the graph represents the enrichment score for each gene set, ranging from 0 to 10. The asterisks indicate signaling pathways shared by the three miRNAs.

To investigate the molecular implications of this group of miRNAs (hsa-miR-182, hsa-miR-760 and hsa-miR-449a) in endometrial cancer patients, their target genes were identified using the online analysis tools miRDB, TargetScan, miRPathDB, and TarBase. A total of 16 overlapping genes were found for hsa-miR-182, 7 overlapping genes for hsa-miR-760 and 39 overlapping genes for hsa-miR-449a (Figure 4A). Next, enrichment analysis was performed to elucidate the biological function of consensus target genes using KEGG pathways. Various cancer-related pathways were significantly enriched in each miRNA. Interestingly, we found that the p53 signaling pathway was common to all of them (Figure 4B). Additionally, by reviewing the gene sets of this pathway, we identified that the *TP53INP1* gene was associated with all three miRNAs. We further analyzed its gene expression and found that it was downregulated in EC samples in contrast to normal tissue samples, as shown in Supplementary Figure S1E.

### 2.5. Two-Dimensional and Three-Dimensional Graphical Representation of the Structure of Hsa-miR-182, Hsa- miR 760, Hsa-miR 449a, and TP53INP1

**Figure 5:**
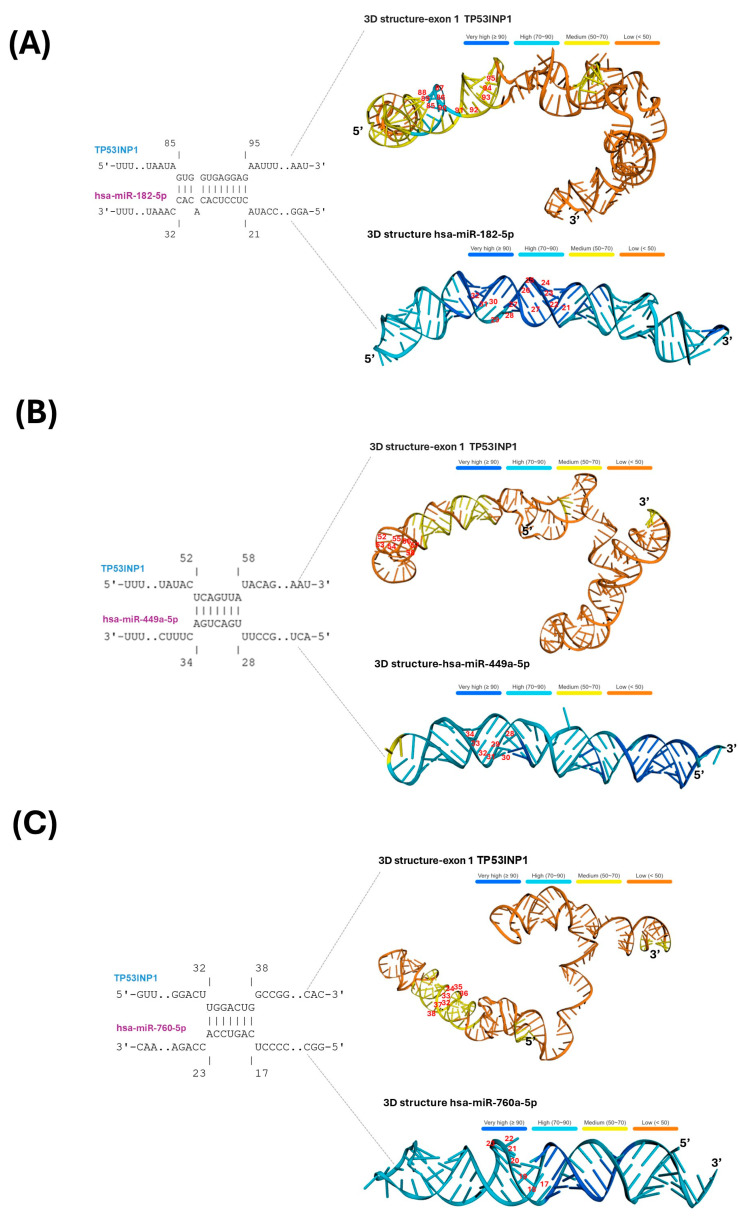
Three-dimensional representation of three microRNAs (miRNAs) and the target gene, *TP53INP1*. Panels (**A**–**C**) display the minimum free energy (MFE) for *TP53INP1* and the miRNAs hsa-miR-182, hsa-miR-760, and hsa-miR-449a, respectively. On the left side of each panel, the specific interaction sites between the miRNAs and their target gene are highlighted. On the right side of each three-dimensional structure, numbers in red indicate the corresponding interaction positions. Additionally, a color bar located at the top of each 3D structure represents the probability of structural formation: dark blue indicates a very high probability (≥90%), light blue a high probability (70–90%), yellow a medium probability (50–70%), and orange a low probability (<50%).

Since the enrichment analysis determined that all miRNAs were related to the p53 signaling pathway and shared *TP53INP1* as a common target gene, we constructed their secondary (Supplementary Figure S1A–C) and tertiary structures (Figure 5A–C) focusing on the specific interaction regions. As shown in Supplementary Figure S1A–C, the 2D loop structuresof the miRNAs had a negative the minimum free energy (MFE), and the nucleotides corresponding to the *TP53INP1* binding sites (highlighted with brackets in each miRNA) appear in red, indicating a high probability of complex formation. Similarly, the 3D structures of the three miRNAs, shown in the lower part of Figure 5A–C, confirmed this high probability of interaction, represented in light blue (probability >70%).

Regarding *TP53INP1*, we identified that the binding sites for hsa-miR-182 are located between nucleotides 85–97 of exon 1, with a predicted interaction probability 70–90%, shown in light blue and yellow regions (Figure 5A). In contrast, the interaction region of hsa-miR-760 showed a probability between 50 and 70%, represented in yellow (Figure 5B), while hsa-miR-449a exhibited the lowest predicted interaction probability (<50%), denoted in orange (Figure 5C).

Additionally, correlation coefficients were calculated between *TP53INP1* expression and these three miRNAs across endometrial cancer samples from stages I to IV. A negative correlation was observed in stage IV between hsa-miR-182 and *TP53INP1* (R = −0.72), as shown in Supplementary Figure S2.

## 3. Discussion

Endometrial cancer is responsible for 42,000 deaths worldwide annually [1]. It is a disease mostly diagnosed after menopause, with type 1 lesions (involving stages I, IIA, and IIB) being the most observed. These lesions are usually hormone-sensitive, low-grade, and have a good prognosis. In contrast, type 2 lesions (involving stages IIC, III, and IV) are high-grade, exhibit a higher recurrence rate, and are associated with poor prognosis [13,14].

Currently, multi-omic studies, such as next-generation sequencing (NGS), are strongly supporting the timely diagnosis and effective treatment of endometrial cancer; for instance, transcriptome analysis provides a detailed understanding of expression changes within the tumor microenvironment [15]. In endometrial cancer, alterations in miRNA expression have been shown to play a crucial role in initiation, progression, and metastasis, through the regulation of adhesion and the cell cycle [16].

In this study, we performed an RNA sequencing analysis on samples from patients with endometrial cancer, obtained from previously published studies by other researchers, to identify differentially expressed miRNAs with a possible association with clinical variables such as diagnosis, prognosis, or relative risk. Although previous studies have reported miRNAs with similar characteristics [9,10], our analysis further explored their involvement in oncogenic signaling pathways to provide deeper insights into their molecular role. Additionally, we calculated the minimum free energy (MFE) of 2D and 3D structures to assess their binding probability to target genes.

Using a discovery cohort of 20 endometrial cancer samples and 11 normal endometrial samples (GSE268888) (Figure 1) and a validation cohort of 504 endometrial cancer samples and 33 normal endometrial samples from the TCGA consortium (Figure 2), we identified that hsa-miR-182, hsa-miR-760, and hsa-miR-449a exhibit consistently high expression in endometrial cancer samples compared to non-neoplasic samples.

Some of these miRNAs have been previously associated with endometrial cancer. Consistent with our findings, Donkers et al. [17] employed the real-time polymerase chain reaction (qPCR) technique and reported an overexpression of hsa-miR-182 in tumor samples compared to non-neoplastic tissues (Figure 2A). However, in contrast to our results (Figure 2A), they identified a higher expression level in advanced disease stages compared to early stages. This discrepancy may stem from their limited sample size, as their study only included eight samples from advanced-stage disease. However, this divergence needs to be experimentally validated in a larger cohort.

Their study also coincided with our findings regarding the diagnostic significance of hsa-miR-182, as reflected by its area under curve (AUC) value of 0.988 (Figure 3D). Interestingly, our findings also highlight that the high expression of this miRNA is significantly associated with a poorer prognosis (Figure 3A).

Regarding the molecular interaction of this miRNA with endometrial cancer, our enrichment analyses (Figure 4B) identified an association between hsa-miR-182 target genes and oncogenic signaling pathways previously linked to endometrial cancer, including EGFR tyrosine kinase inhibitor resistance, which, when inhibited by the enzyme fumarate hydratase, leads to a decrease in proliferation and metastasis [17]; the tumor suppressor FoxO, which shows reduced expression in endometrial cancer samples compared to healthy tissues [18]; and the PI3K-Akt pathway [19], which is dysregulated in this cancer type and contributes to therapeutic resistance.

Regarding hsa-miR-760, its overexpression in endometrial cancer and its relationship with the lower probability of overall survival aligns with the findings of Hegde et al. [12]. Additionally, our study revealed that its overexpression is associated with an increased risk of developing the disease (Figure 3C) and has a diagnostic significance supported by an AUC value of 0.790 (Figure 3D), a finding that had not been previously reported. Likewise, we identified hsa-miR-760, through its target genes, as being related to oncogenic pathways strongly associated with the development and maintenance of cancer, such as protein processing in the endoplasmic reticulum, as both the increased expression of glucose-regulated protein 78 (GRP78) and endoplasmic reticulum stress have been shown to promote growth and invasion of endometrial cancer cells [20], and transcriptional misregulation in cancer [21], which may be linked to epitranscriptomic modifications mainly affecting ribosomal and transfer RNAs. Furthermore, hsa-miR-760 was associated with the ATP-dependent chromatin remodeling pathway [22], as these enzymes play a causal and facilitating role in the progression to advanced-stage cancers (Figura 4B).

The last miRNA that maintained overexpression in our validation cohort was hsa-miR-449a, which coincides with the findings reported by Hegde et al. [12]. Additionally, our results suggest that hsa-miR-449a exhibits a significant increase in expression at stage I, but this pattern is not maintained in stage II or in more advanced stages (III, IV) (Figure 2C). On the other hand, hsa-miR-449a did not show a significant association with survival or disease risk (Figure 3C), but it demonstrated diagnostic relevance, supported by an AUC of 0.87 (Figure 3D). These findings suggest that this miRNA could be related to the initiation of endometrial cancer rather than its progression; however, further studies are needed to validate this hypothesis.

In terms of molecular regulation, hsa-miR-449a was also associated with signaling pathways involved in oncological processes, including “MicroRNAs in cancer”, “Pathways in cancer”, and “Chemical carcinogenesis”. Interestingly, we also found positive enrichment in the “Breast Cancer pathway”, which is another type of neoplasm that primarily affects women. In this context, the role of hsa-miR-449a is controversial, as it has been shown to mediate chemoresistance in the triple-negative subtype [23]; however, other studies have also reported that it can inhibit tumor progression through DNA repair mechanisms [24]. Therefore, it is necessary to clarify whether, in the context of endometrial cancer, it could also present functional heterogeneity.

The most relevant finding of this study was the identification that, although some signaling pathways showed a common positive enrichment for the target genes of the three miRNAs described, p53 signaling pathway exhibited positive enrichment for all miRNAs (Figure 4B). Additionally, it was identified that among the genes belonging to this pathway, *TP53INP1* (tumor protein p53-inducible nuclear protein 1) is a common target gene of hsa-miR-182, hsa-miR-760, and hsa-miR-449a. Interestingly, the probability of complex formation is high, according to the 2D (Supplementary Figure S1) and 3D (Figure 5A–C) structural predictions conducted in this study.

Although this study does not provide a mechanistic explanation for the findings, we include a boxplot (Supplementary Figure S1) showing *TP53INP1* expression, as well as a correlation analysis (Supplementary Figure S2) between *TP53INP1* and hsa-miR-182. These figures show that *TP53INP1* exhibits lower expression in endometrial cancer samples compared to normal endometrial tissues, and that its correlation with hsa-miR-182 is significantly negative (R = −0.72) in stage IV of the disease. These results, together with the high expression of hsa-miR-182 in tumor samples compared to non-tumor samples (Figure 2A), allow us to suggest that although all three miRNAs described in this study show a high binding probability to *TP53INP1*, hsa-miR-182 may exert a stronger influence on the regulation of its expression in the context of this disease. Nevertheless, these findings highlight the need for further research to confirm whether *TP53INP1* repression is directly linked to the overexpression of these miRNAs.

Additionally, this study may be relevant for future functional studies, particularly in the development of RNA interference (RNAi) aimed at disrupting the interaction between these miRNAs and *TP53INP1*. This approach would allow us to assess whether such inhibition results in an increase in *TP53INP1* expression, and whether it impacts the onset and/or progression of endometrial cancer. Lastly, we highlight as the main limitation of this study, the inability to validate our findings in samples derived from patients with endometrial cancer, an aspect we plan to address in future investigations to strengthen the applicability of our findings.

## 4. Materials and Methods

### 4.1. DEMs Analysis in Endometrial Cancer Dataset

To determine the differentially expressed miRNAs (DEMs), we analyzed a database comprising 11 cancer-free and 20 EC-derived samples, available in the GEO NCBI repository (https://www.ncbi.nlm.nih.gov/gds (accessed on 10 September 2024)) under accession code GSE268888 [12]. Some of the samples available in this dataset were excluded from the analysis because they did not meet the criteria for classification as tumor or non-tumor at the time of performing the principal component analysis (PCA). For the bioinformatics analysis, we compared these groups using the Galaxy Europa open-source platform (usegalaxy.eu) and R version 4.1.2 (available at https://cran.r-project.org/bin/windows/base/old/, last accessed on 10 October 2024), RStudio software (2021.09.0) (available at https://dailies.rstudio.com/version/2021.09.0+351/, last accessed on 20 October 2024), utilizing Rsubread library.

Quality control was established with the FastQC tool (version 0.73 with galaxy0) [25]; then, ambiguous nucleotides detected were removed using Trimmomatic tool (version 0.38.1) [26]. Clean reads were then aligned to the Human genome package Version hg38 (vs. 38) using Rsubread, generating BAM files. These files were used to obtain count reads with the featureCounts tool (version 2.0.1 with galaxy2) [27].

Differential expression analysis was performed using DESeq2 (version 2.11.40.7 with galaxy1) [28]; FPKM (fragments per million kilobases) was used for normalization. The volcano Plot and HeatMap in Figure 1A, B were generated with the packages Enhanced Volcano [29], and heatmaps [30], available from Bioconductor (available at https://bioconductor.org/, last accessed on 20 October 2024). Genes were considered differentially expressed if they had a Fold Change ≥ 1.5 or ≤1.5, with statistical significance at an adjusted *p*-value 0.05.

### 4.2. Expression of DEMs in Endometrium from the TCGA Dataset

To validate our findings, we extended our analysis to DEMs with high expression levels, utilizing data from 504 samples EC from the TCGA database (available at https://portal.gdc.cancer.gov, last accessed on 20 October 2024). An independent comparative analysis was then conducted between normal endometrial samples and each EC molecular subtype, specifically the 317 subtype I, 44 subtype II, 118 subtype III, and 25 subtype IV samples. For the analyses described below, we used R version 4.1.2 (https://cran.r-project.org/bin/windows/base/old/, last accessed on 20 October 2024) and RStudio software version 2021.09.0 (https://dailies.rstudio.com/version/2021.09.0+351/, last accessed on 20 October 2024).

Differential expression analysis was performed using the edgeR package (v 3.40.2) from Bioconductor (https://bioconductor.org/packages/release/bioc/html/edgeR.html, last accessed on 20 October 2024) [31], based on miRNA expression data obtained from the htseq-counts files. The screening criteria used were |logFold Change| > 1.5, and a false discovery rate (FDR) < 0.05.

For the construction of the boxplot plots in Figure 2A–C, the nonparametric Wilcox test was applied, and it was created using the ggpubr package, version 0.6.0 (available at https://cran.r-project.org/web/packages/ggpubr/index.html, last accessed on 20 October 2024).

### 4.3. Overall Survival, Univariate Cox Regression, and ROC Analysis

Risk scores were calculated based on normalized expression levels, applying the log-rank test and Cox regression analysis to assess the statistical significance of differences between high- and low-expression groups for each of the overexpressed DEMs. For this analysis, we used the online tool KMPlot, which is available at https://kmplot.com/analysis/index.php?p=home, last accessed on 1 November 2024.

To verify the discriminatory ability of the identified DEMs to separate samples without EC from samples with EC, we analyzed their expression values, calculated sensitivity and specificity parameters, and generated receiver operating characteristic (ROC) curves. The package used to construct the ROC curves shown in Figure 3D was the pROC package (available at https://cran.r-project.org/web/packages/pROC/pROC.pdf, last accessed on 12 November 2024) [32]. All expression values were obtained from endometrial cancer samples from the TCGA database.

### 4.4. Target Gene Prediction of DEMs

The target genes of overexpressed DEMs validated in the TCGA cohort were predicted using TargetScan (available at http://www.targetscan.org/, last accessed on 1 December 2024), miRDB (available at https://mirdb.org/, last accessed on 1 December 2024), miRPathDB (available at https://mpd.bioinf.uni-sb.de/, last accessed on 1 December 2024), and TarBase (available at https://www.mirnet.ca/, last accessed on 9 April 2025).

To enhance the reliability of bioinformatics predictions, we identified overlapping target genes using Venn diagram analysis, employing the Bioinformatics and Evolutionary Genomics tool (available at https://bioinformatics.psb.ugent.be/webtools/Venn/, last accessed on 3 December 2024).

Subsequently, we identified the specific interaction sites between hsa-miR-182, hsa-miR-760, and hsa-miR-449a and their target genes within the positively enriched signaling pathway, particularly P53 (including *TP53INP1*). This analysis was performed using the IntaRNA-RNA interaction online tool (https://rna.informatik.uni-freiburg.de/IntaRNA/Input.jsp?example=IntaRNA_57244, last accessed on 10 December 2024).

Briefly, the IntaRNA algorithm predicts RNA-RNA interactions by integrating seed constraints and site accessibility parameters. Detailed mathematical methods can be found in the study by Mann M and Wright R [33] (available at https://rna.informatik.unifreiburg.de/IntaRNA/Input.jsp?example=IntaRNA_57244, last accessed on 10 December 2024).

### 4.5. Enrichment Analysis

For enrichment analysis, the overlapping target genes of hsa-miR-182, hsa-miR-760, and hsa-miR-449a were analyzed using The Database for Annotation. The GENECODIS online tool (last accessed on 2 February 2025, https://genecodis.genyo.es/) was employed for this purpose. The analyses were performed using KEGG pathway data, applying the Hypergeometric method for enrichment, and considering only evidence with experimental annotation. Pathways were considered significant if they had an adjusted *p*-value (P-Adj) ≤ 0.05. The pathways with the highest positive enrichment scores were selected for inclusion in Figure 4.

### 4.6. Two-Dimensional and Three-Dimensional Structure Topology Prediction

To determine the probability of interaction region formation between hsa-miR-182, hsa-miR-760, and hsa-miR-449a and their target *TP53INP1*, we used variant 1, in the National Library of Medicine (accession code NM_033285.4, available at https://www.nlm.nih.gov/, last accessed on 10 January 2024). For 2D structure prediction, individual sequences were analyzed using RNAfold included in the ViennaRNA package [34]. The most thermodynamically stable structures, characterized by minimum free energy (MFE), were selected. The mathematical description of the algorithm used is described in detail in the work of Zuker and Stiegler [35], and RNAfold is available in the online tool BioAlphaFold (available at http://rna.tbi.univie.ac.at//cgi-bin/RNAWebSuite/RNAfold.cgi, last accessed on 10 January 2024) [36].

Subsequently, the 2D models obtained for each miRNA (Supplementary Figure S1) and their target genes were used to construct 3D structures using the deep learning algorithm trRosettaRNA, which enhances prediction reliability through blind tests (including CASP15 and RNA-Puzzles). The algorithm selects the structure with the lowest MFE (available at https://yanglab.qd.sdu.edu.cn/trRosettaRNA/, last accessed on 21 January 2024). Figure 5 presents the specific interaction between each miRNA and its respective target gene *TP53INP1*.

### 4.7. Correlation Analysis

The correlation analyses between *TP53INP1* and hsa-miR-182, (Supplementary Figure S2), was performed using expression data normalized as counts per million (CPM), obtained from the TCGA consortium. Pearson correlation was applied for the statistical analysis, considering *p*-values < 0.05 as statistically significant. The calculation of correlation coefficients and statistical significance was carried out using R version 4.1.2 (https://cran.r-project.org/bin/windows/base/old/, last accessed on 9 April 2025) and RStudio software version 2021.09.0 (https://dailies.rstudio.com/version/2021.09.0+351/, last accessed on 9 April 2025).

## 5. Conclusions

In this study, we identified a miRNA signature composed of hsa-miR-182 and hsa-miR-760, with elevated expression in endometrial cancer samples compared to non-tumoral samples. hsa-miR-182 and hsa-miR-760 demonstrated both diagnostic and prognostic relevance, while hsa-miR-449a showed diagnostic utility. Functional enrichment analyses associated this miRNA signature with oncogenic signaling pathways, particularly highlighting the p53 signaling pathway, in which all three miRNAs shared *TP53INP1* as a common target gene.

The high probability of interaction between these miRNAs and *TP53INP1* suggests a potential downregulation of this gene’s expression, especially by hsa-miR-182, which exhibited a significant negative correlation with *TP53INP1* expression in stage IV of the disease.

These findings may be relevant for future studies aiming to evaluate whether modulation of this interaction can influence the onset or progression of endometrial cancer. Additionally, the analyzed miRNAs may have potential as clinical biomarkers. However, further experimental validation is required to confirm these results and their clinical applicability.

## Data Availability

The datasets analyzed in this study were obtained from the public database of the Cancer Genome Atlas Program (TCGA), accessible at https://www.cancer.gov/ccg/research/genome-sequencing/tcga (accessed on 20 October 2024), and from the study with the accession code GSE268888, available in the GEO NCBI Repository (https://www.ncbi.nlm.nih.gov/gds (accessed on 20 October 2024)).

## Supplementary Materials

The following supporting information can be downloaded at: https://www.mdpi.com/article/10.3390/ijms26115215/s1.

## Abbreviations

The following abbreviations are used in this manuscript:

EC	endometrial cancer
DEMs	differentially expressed miRNAs
TCGA	The Cancer Genome Atlas
CPM	counts per million
Log2FC	log-transformed gene expression change with base 2
AUC	area under the curve
ROC	receiver operating characteristic
HR	hazard ratio
MFE	minimum free energy
NGS	next-generation sequencing
qPCR	real-time polymerase chain reaction
FPKM	fragments per million kilobases
