# Supplementary material for: Alterations in the Expression of a Set of miRNAs in Endometrial Cancer and Their Correlation with Clinical Variables and the p53 Signaling Pathway
Source: Int J Mol Sci. 2025 May 29;26(11):5215. doi: 10.3390/ijms26115215 (PMC12155133; doi:10.3390/ijms26115215)
Supplement: Supplementary file 1 [file ijms-26-05215-s001.zip › Supplementary Figure 1.pdf]

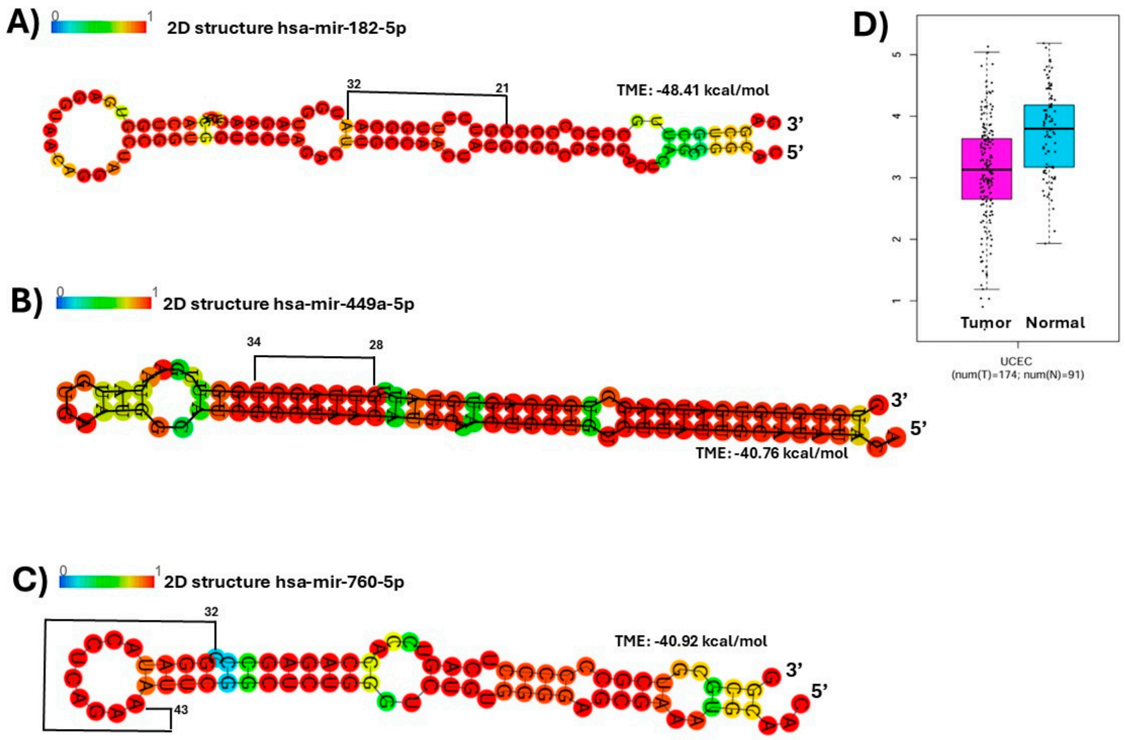

Supplementary Figure 1, Representation of the Loop Secondary Structure of three miRNAs and *TP53INP1* Expression. Panels (**A**), (**B**), and (**C**) represent the minimum free energy (MFE), with base pair probabilities shown in different colors for each nucleotide. Red pairs have a high probability of forming, green pairs have a medium probability, and blue pairs have a low probability. The normalized scale, ranging from zero (dark blue) to one (red), is displayed in the upper left corner. The figure was generated using the ViennaRNA Websuite with default parameters. Black brackets indicate the binding site of each miRNA to the *TP53INP1* gene. TME: thermodynamic set free energy. E) *TP53INP1* gene expression in tumor-derived and normal samples, represented as Log2(TPM + 1) on a logarithmic scale.
