# Supplementary material for: Alterations in the Expression of a Set of miRNAs in Endometrial Cancer and Their Correlation with Clinical Variables and the p53 Signaling Pathway
Source: Int J Mol Sci. 2025 May 29;26(11):5215. doi: 10.3390/ijms26115215 (PMC12155133; doi:10.3390/ijms26115215)
Supplement: Supplementary file 1 [file ijms-26-05215-s001.zip › Supplementary Figure 2.pdf]

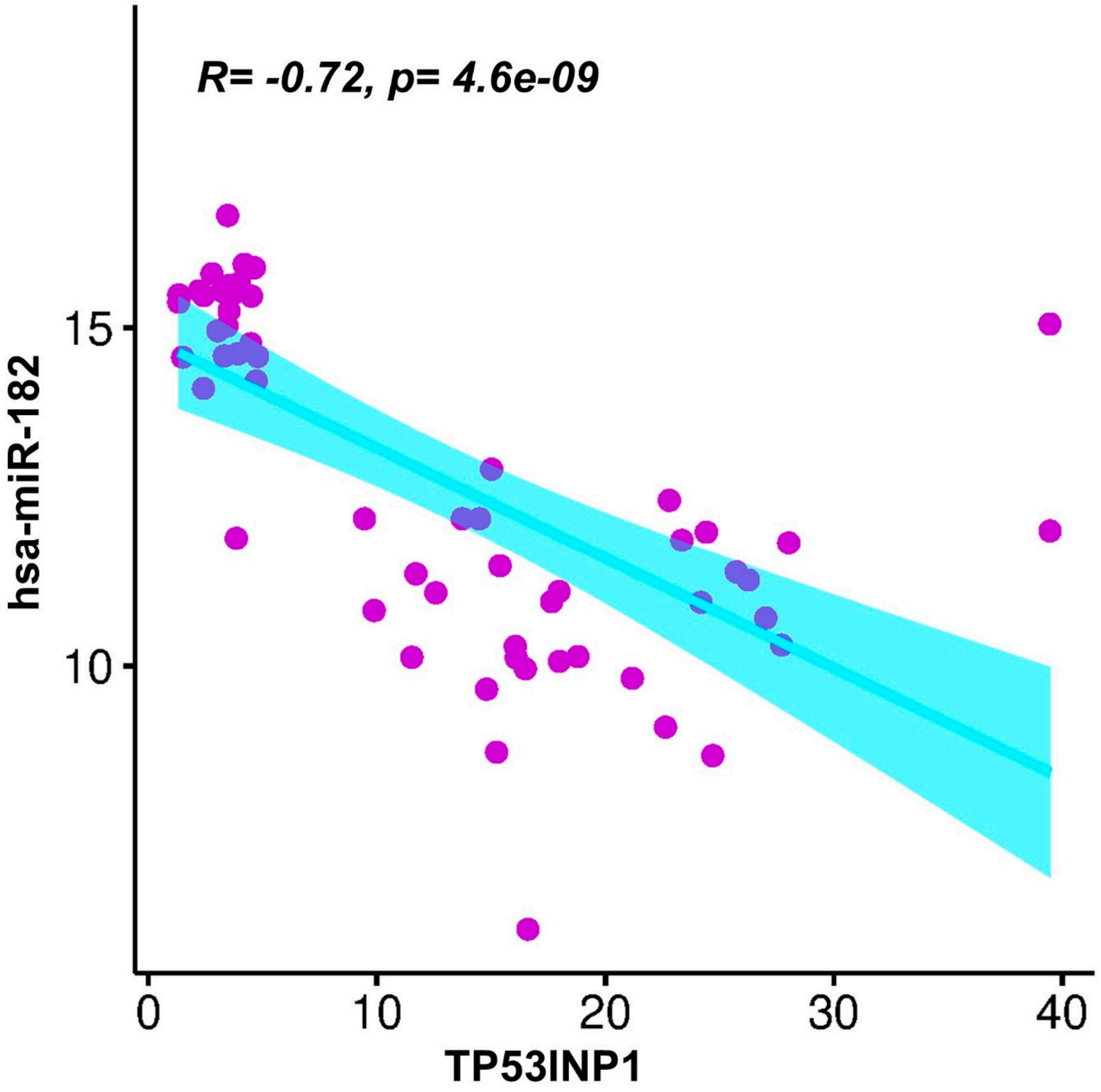


**Supplementary Figure 2, Scatter plot showing the Pearson correlation coefficients between *TP53INP1* and hsa-miR-182 expression in clinical stage IV endometrial cancer.** The Pearson correlation coefficient (R) is indicated. Fuchsia-colored dots represent the expression levels for individual patients.
