# Supplementary material for: Alterations in the Expression of a Set of miRNAs in Endometrial Cancer and Their Correlation with Clinical Variables and the p53 Signaling Pathway
Source: Int J Mol Sci. 2025 May 29;26(11):5215. doi: 10.3390/ijms26115215 (PMC12155133; doi:10.3390/ijms26115215)
Supplement: Supplementary file 1 [file ijms-26-05215-s001.zip › Supplementary Table 1.pdf]

Supplementary Table 1. The minimum residual energy of the interaction between miRNAs and *TP53INP1*."

**miRNA / Position**	***TP53INP1*/ Position**	**Hybridization energy**
**hsa-mir-185-5p/**21-32	85-95	-18.34
**hsa-mir-449a-5p/**28-34	52-58	-7.28
**hsa-mir-760a-5p/**17-23	32-38	-9.77
